# Effort responses to suboptimal reward cues are related to striatal dopaminergic functioning

**DOI:** 10.1007/s11031-014-9434-1

**Published:** 2014-10-07

**Authors:** Pascal Pas, Ruud Custers, Erik Bijleveld, Matthijs Vink

**Affiliations:** 1 University Medical Center Utrecht, Utrecht, The Netherlands; 2 Experimental Psychology, University College London, 26 Bedford Way, London, WC1H 0AP UK; 3 Utrecht University, Utrecht, The Netherlands; 4 Behavioural Science Institute, Radboud University Nijmegen, Nijmegen, The Netherlands

**Keywords:** Rewards, Motivation, Reward-sensitivity, Subliminal priming, ERP

## Abstract

Reward cues have been found to increase the investment of effort in tasks even when cues are presented suboptimally (i.e. very briefly), making them hard to consciously detect. Such effort responses to suboptimal reward cues are assumed to rely mainly on the mesolimbic dopamine system, including the ventral striatum. To provide further support for this assumption, we performed two studies investigating whether these effort responses vary with individual differences in markers of striatal dopaminergic functioning. Study 1 investigated the relation between physical effort responses and resting state eye-blink rate. Study 2 examined cognitive effort responses in relation to individually averaged error-related negativity. In both studies effort responses correlated with the markers only for suboptimal, but not for optimal reward cues. These findings provide further support for the idea that effort responses to suboptimal reward cues are mainly linked to the mesolimbic dopamine system, while responses to optimal reward cues also depend on higher-level cortical functions.

## Introduction

People generally invest more effort in demanding tasks if the anticipated rewards are more valuable (Brehm and Self 1989; Gendolla et al. 2012). As such, cues that indicate that valuable rewards are at stake increase effort expenditure during a wide variety of physical and cognitive tasks. These effort responses are generally considered to result from a deliberate decision making process, in which people weigh the pros and cons of potential effortful actions. In other words, people are thought to weigh the value of anticipated rewards (the benefits) against their respective effort requirements (the costs). Challenging this traditional perspective on human motivation, a recent series of studies has shown that reward cues can instigate effortful behavior even when these cues are presented suboptimally (i.e. very briefly), which makes conscious processing less likely (see for reviews Bijleveld et al. 2012b; Custers and Aarts 2010). So, the expenditure of effort may be initiated without deliberate decisions.

At this point, the neurobiological mechanisms that underlie this intriguing phenomenon are still subject to debate. In the present research, we explore the idea that effort responses to suboptimal reward cues are dependent on dopaminergic activity in the ventral striatum. Specifically, we use two physiological correlates of such activity—resting state eye-blink rate (EBR, Study 1) and error-related negativity (ERN, Study 2)—to examine the relation between effort responses and dopaminergic functioning. In doing so, we hope to further our understanding of how reward cues influence effort expenditure in the absence of deliberate decisions.

### Effort responses to suboptimal reward cues

Pessiglione et al. (2007) first demonstrated that suboptimal reward cues can affect the expenditure of effort even when people cannot consciously discriminate between high and low reward cues. In their experiment, participants could earn money by squeezing forcefully into a handgrip. At the beginning of each trial of their experiment, a coin was presented that represented the amount of money that was at stake. This coin was either one British pound (high reward) or one penny (low reward). The harder participants squeezed the handgrip, the higher the proportion of the presented reward they received. Remarkably, participants not only squeezed harder on high versus low reward trials when the coins were presented long enough to be clearly visible, but also when they were presented too briefly to allow conscious discrimination. For optimal reward cues, functional MRI data showed significant brain activation for high (vs. low) reward cues (see also Van Hell et al. 2010), especially in the ventral striatum and ventral pallidum, which are output channels of the striatal dopamine system (Heimer and Van Hoesen 2006). However, although there was a significant increase in the effort people invested in the task for high versus low reward cues, no significant differences in brain activation were found for suboptimal reward cues. Hence, the neurobiological basis of the behavioral consequences of suboptimal reward cues is still unclear.

Using behavioral paradigms, though, a considerable number of studies have found reliable effects of suboptimal reward cues on effortful behavior (see for an overview Capa and Custers 2013). Although the effects on task performance are often similar to those generated by optimal reward cues (e.g., Bijleveld et al. 2009), recently researchers have started to study the circumstances under which the effects of optimal and suboptimal reward cues diverge. Bijleveld et al. (2010), for instance, looked at speed-accuracy trade-offs in a math task in which the percentage of the cued reward that was earned for correct answers declined with time. Suboptimal high (vs. low) reward cues were found to increase speed without affecting accuracy. Optimal reward cues caused a slowdown, but an increase in accuracy. Whereas optimal reward cues clearly altered behavior through strategic processes (i.e. slowing down and thus sacrificing a little bit of the reward in order to be more accurate), suboptimal reward cues seemed to only boost the effort people exerted on the trial. Such dissociations have been found in various other studies (e.g., Zedelius et al. 2011, 2012), suggesting that optimal and suboptimal reward cues affect the investment of effort through processes that are (at least partly) distinct.

To account for these findings, Bijleveld et al. (2012b) have proposed that responses to reward cues can be understood by distinguishing two phases in the processing of reward cues. Initially, rewards are valuated and processed by rudimentary brain structures, which may occur without conscious awareness. As a consequence of such initial processing, effort is increased (but only if the task demands it, Bijleveld et al. 2009), which in turn facilitates performance. This initial reward processing is thought to rely on the mesolimbic dopamine system, including the ventral striatum, which supports effort responses to rewards in animals that lack the cortical sophistication that is characteristic of humans (e.g., Phillips et al. 2007). If a reward cue is presented long enough, however, the reward is thought to undergo full reward processing which enables deliberate decision making. In this case, information carried by the reward cue becomes available to higher-order brain functions, presumably located in the cortex. Associated with conscious deliberation, these higher-order functions enable strategic reward-related decision-making processes and enable people to reflect on the reward that is at stake. Such higher-order functions can affect performance beyond the mere expense of effort. For example, they change tradeoffs in speed versus accuracy (Bijleveld et al. 2010), change the way attention is deployed to task stimuli (Bijleveld et al. 2011), and induce people to disengage from tasks altogether when the payoff is deemed too small (Bijleveld et al. 2012a).

Based on the theoretical framework laid out above, it can be hypothesized that activity in the ventral striatum is especially predictive of performance when reward cues are presented suboptimal and only processed initially (unless there is less room for strategic behavior, e.g., Pessiglione et al. 2007). After all, we suggest that initially processed rewards directly boost the expenditure of effort in demanding tasks, making use of only this subcortical infrastructure (and not of strategic functions located in the cortex). In the present paper, we test this idea. Specifically, we investigate whether individual differences in striatal dopaminergic functioning predict performance due to initially processed reward cues.

### Striatal dopaminergic functioning

The ventral striatum has been identified as a key structure in the processing of reward cues by studies showing that activity in the striatum was correlated with the value of the rewards at stake (Bjork and Hommer 2007; Knutson and Greer 2008; Schultz et al. 1997). The striatum connects to various other structures, such as the pallidum, that are implicated in goal-directed behavior (Aston-Jones and Cohen 2005; Knutson et al. 2008) and has been linked to reward prediction during learning tasks (O’Doherty et al. 2004; Pessiglione et al. 2007). These findings are in line with research on rodents, demonstrating that pallidal and striatal neurons encode for rewarding properties of environmental stimuli (Tindell et al. 2006).

Key to the activity in these reward centers of the brain is the neurotransmitter dopamine (Björklund and Lindvall 1984). Stimuli signaling potential rewards as well as the presence of rewards have been found to trigger dopamine release in the striatum, which respectively increases effort directed at reward attainment and learning of stimulus-reward contingencies (Schultz 2006). Although the way in which dopamine influences reward-directed effort is not yet fully understood (Braver et al. 2014), it appears that general levels of dopamine in the striatum have to be taken into account to predict effects on motivation. That is, apart from phasic shifts in dopamine, tonic dopamine levels seem to affect the overall vigor with which rewards are pursued. These levels are affected by recently encountered rewards, general motivational states such as thirst and hunger, but also individual differences in baseline dopamine levels (Niv et al. 2007). In rats, striatal dopamine depletion (i.e. low baseline level) has been found to be associated with a lack of reward pursuit, especially when pursuit is effortful (Phillips et al. 2007). Baseline striatal dopamine levels, then, may be related to general dopaminergic functioning and moderate the effect of reward cues on effort responses. Therefore, if effort responses to suboptimal reward cues are dependent on initial processing in the striatum, these effort responses to these reward cues are likely to be correlated with striatal dopaminergic functioning.

To test this hypothesis, we present two studies in which we investigate whether effort responses to reward cues are related to two different markers of striatal dopamine functioning: EBR and ERN. In Study 1, we investigated if resting state EBR is related to physical effort responses in reaction to suboptimal reward cues. In Study 2, we used another marker of striatal dopamine functioning, individually averaged ERN, and investigated whether this measure is related to mental effort.

## Study 1

Resting state EBR is strongly linked to activity in the striatal dopamine system (Karson 1983). Investigating Parkinson patients that are characterized by low EBRs and low levels of striatal dopamine, Karson showed that patients receiving levodopa medication (a dopamine agonist) exhibited twice the mean EBR of that of other Parkinson patients. Furthermore, the more symptomatic patients of the non-levodopa group showed significantly lower blink rates. Other clinical observations show elevated EBR in patients with increased levels of dopamine in the striatum, including symptomatic schizophrenic patients (Howes and Kapur 2009; Kegeles et al. 2010).

Importantly, EBR has been found to be correlated with personality traits such as impulsivity, novelty seeking, and positive emotionality, which in turn are associated with reward sensitivity (Dagher and Robbins 2009; Depue et al. 1994; Huang et al. 1994; Martin and Potts 2004). For instance, impulsive individuals tend to prefer immediate rewards, and choosing immediate rewards is associated with greater activity in areas innervated by the mesolimbic dopamine system, including the ventral striatum (Hariri et al. 2006; McClure et al. 2004). If effort responses to suboptimal reward cues indeed rely on striatal dopaminergic functioning, these responses should increase with EBR.

To examine the effects of reward cues on physical effort, we relied on a task that was used by Bijleveld et al. (2012a). In line with the research of Pessiglione et al. (2007), participants in this task are on each trial presented with either a high or low value coin that is displayed either for a brief or a long time interval. After the presentation of the reward cue, participants have to repeatedly press a button within a specific time limit in order to obtain the reward. We expected to find a reward effect (i.e. participants expending more effort in the high reward, 10-cent trials, than in the low reward, 1-cent trials) for both optimal and suboptimal reward cues, but that the occurrence of this effect would correlate positively with individual differences in EBR only for suboptimal cues.

### Methods

#### Participants

Forty-one (33 female) healthy undergraduate and graduate students (*M*_age_ = 21.24; *SD* = 1.61) participated in the study for a financial reward. Several selection criteria were applied (Colzato et al. 2009): All participants were healthy volunteers, and reported no psychiatric or neurologic disorders, nor brain trauma. Furthermore, participants declared not to use drugs or psychoactive medication, and did not smoke. In addition, per request participants did not consume any beverages on the day of the study that contained caffeine.

#### EBR measurement

EBR was recorded by using infrared videography technology (Tobii X120 Eye Tracker, 120 Hz sampling rate). Participants were asked to sit in a chair, in upright position, and look straight ahead for 5 min at a white fixation cross (35 by 35 mm) on a black screen (60 Hz LCD monitor with a resolution of 1,024 by 768, at a distance of 60 cm). An infrared eye tracker underneath the screen registered the participants’ eye blinks. After the actual thirty-second calibration, participants were told calibration of the eye tracker would continue for another 5 min, during which blinks where measured. A blink was defined using the points of missing data from both eyes between 100 and 500 ms (Aarts et al. 2012). After the removal of three outliers (>2 *SD* above the mean, with an unrealistically high EBR above 55 blinks/min suggesting artifacts from the equipment) and two participants suffering from hay fever, mean EBR per minute in our sample was 18.92 (*SD* = 10.38).

#### Experimental task

To assess the reward priming effect we used a ‘finger-tapping task’ in which effort expenditure was measured (Bijleveld et al. 2012a; see also Treadway et al. 2009), and this task was started directly after the EBR measurement. On each trial, participants needed to execute a specific amount of button presses (25) within a set time (3.5 s) in order to obtain a monetary reward. Finishing the button presses outside of the time limit was allowed, but simply did not lead to obtaining the reward. Each button press filled an empty circle in a bar on the screen, so that after each button press the bar filled up providing feedback. Participants received money when they successfully filled the bar within the time limit. Each trial started with a fixation cross displayed on the screen for 1,000 ms, followed by a 300 ms pre-mask, either the high or low value stimulus displayed long (300 ms) or brief (17 ms). The duration of the post mask was either 483 or 200 ms, to keep the total duration of the stimulus presentation constant. After a 1,000 ms fixation cross the response screen was visible. Trials ended with a feedback screen displaying whether the participant was fast enough in order to obtain the reward. This reward was equal to the value of the coin that was presented at the beginning of the trial. To prevent participants from using two hands, each trial was started by holding a key with the non-dominant hand while the dominant hand remained free to execute the button presses.

The study used a 2 (reward value: 1 vs. 10 cents) × 2 (reward presentation: suboptimal vs. optimal) within-subjects design. Participants started with 16 practice trials in order to get familiar with the task demands, and these were identical to the actual task but participants did not receive the money earned. The actual task thereafter consisted of 64 trials. Trials were presented in random order.

#### Reward cues

Following the exact same stimuli and procedure for reward presentation as Bijleveld et al. (2012a), the picture of 10 a Eurocent coin was taken as a high reward cue and the picture of a 1 Eurocent coin as a low reward cue. The pictures depicting the coin were 120 × 120 pixels in size and were presented for a brief (17 ms) or long (300 ms) time interval between masks. The suboptimal presentation time in combination with the masking proved too fast to allow conscious discrimination above change level in a standard signal detection task in previous research (Bijleveld et al. 2012a).

### Results and discussion

#### Reward effect

The median tapping times (i.e. the time it took to press 25 times) were subjected to analysis of variance, with reward value (low vs. high) and reward presentation (suboptimal vs. optimal) as within-subjects variables. This analysis revealed a significant main effect of reward value, *F*(1, 35) = 24.21, *p* < .001, η_*p*_^2^ = .41; reward presentation, *F*(1, 35) = 10.19, *p* = .003, η_*p*_^2^ = .23; and the interaction effect, *F*(1, 35) = 21.25, *p* < .001, η_*p*_^2^ = .38. The reward effect was stronger for optimal, *t*(36) = 4.78, *p* < .001 (one-tailed; *M*_low_ = 3,775, *SD* = 837; *M*_high_ = 3,102, *SD* = 281) than for suboptimal cues, *t*(36) = 2.03, *p* = .02 (one-tailed; *M*_low_ = 3,222, *SD* = 337; *M*_high_ = 3,198, *SD* = 315), although for both presentation times, high reward cues yielded significantly faster tapping than low reward cues. This difference seems to be mainly driven by participants refraining from investing effort on optimal low reward trials (cf., Bijleveld et al. 2010).

#### Eye blink rate

**Fig. 1:**
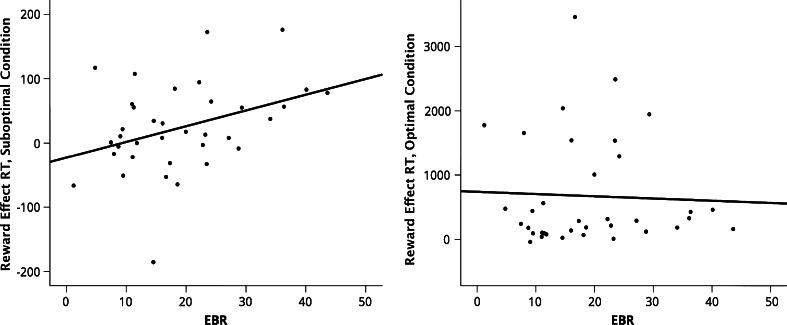
Study 1: Scatterplots of the reward effect against baseline EBR in both the suboptimal condition (*left*) and the optimal condition (*right*), for response times (performance on low reward trials subtracted from high reward trials)

A Pearson correlation was computed to test for a link between EBR and the reward effect, which was calculated by subtracting the tapping times of the high reward value trials from those on the low reward value trials so that higher values indicate a speed up for higher rewards. The test revealed a positive correlation for suboptimal reward cues, *r*(36) = .37, *p* = .02 (one-tailed), but not for optimal reward cues *r*(36) = −.04, *p* = .81 (see Fig. 1). The difference between these two correlations proved significant, with a Steiger’s Z score of 1.73, *p* = .02 (one-tailed; Steiger 1980).

These results first of all show that participants indeed exerted significantly more effort when presented with the high value coin, regardless of whether this presentation was optimal or suboptimal. Secondly, a significant correlation between participants’ EBR and their reward effect on performance was found for suboptimal, but not for optimal reward cues. In conclusion, assuming EBR is a representative marker, the results of this first study are suggesting a connection between striatal dopaminergic functioning and people’s effort responses to reward cues, but only when these are presented suboptimally.

## Study 2

To increase convergent validity, we opted for a different marker of individual differences in striatal dopamine activity in Study 2. An important potential drawback of EBR is that it cannot be measured at the actual time of the experimental task. To overcome this drawback, Study 2 used ERN instead of EBR, a neurophysiological measure that can be measured simultaneously with task performance. Moreover, we used a cognitive task instead of a physical task, where response accuracy and speed served as indicators of performance. In previous research, cognitive tasks have successfully been used to discern between the effects of optimal and suboptimal reward cues, both on the behavioral and the brain level (e.g., Bijleveld et al. 2010; Capa et al. 2012).

Electroencephalography was used to obtain event-related potentials (ERP) during the task. In the last decade, studies have identified a neural response to errors that has been termed the ERN or error negativity. Observed at fronto-central recording sites, ERN consists of a large negative shift in the response, or feedback-locked ERP occurring 50–100 ms after subjects have made an erroneous response (Holroyd and Coles 2002). Localization with dipole localization algorithms has led most researchers to conclude that ERN originates in the anterior cingulate cortex, a structure directly connected to the ventral striatum.

There are several indications that the amplitude of the ERN can be interpreted as a correlate of functioning of the dopamine system. For example, previous studies show that administering drugs that increase striatal dopaminergic activity, such as amphetamine or caffeine, also increase ERN. Moreover, patients with schizophrenia, obsessive–compulsive disorder and Parkinson’s disease, all characterized by disturbances in dopaminergic activity, show abnormal ERNs (Holroyd et al. 2002; Olvet and Hajcak 2008). Similarly, ERN is observed to decline together with weakened dopaminergic activity accompanying old age (Nieuwenhuis et al. 2010). This leads us to suggest that taking average ERN over all incorrect trials reflects dopaminergic functioning in participants, and subsequently yields a meaningful individual difference measurement.

### Methods

#### Participants

Thirty participants were recruited (*M*_age_ = 21.53, *SD* = 2.40, 25 females), via flyers distributed at university buildings. Subject screening was done through an online questionnaire to ensure they met the same inclusion criteria as in the previous study. One subject declared after the study to have been diagnosed with ADHD, and was therefore left out of the analysis. Another subject was left out due to equipment failure, bringing the final amount to twenty-eight (*M*_age_ = 21.64, *SD* = 2.45, 23 females). Participants were compensated with the money they earned during the study, which was on average €12.92.

The study used a 2 (reward value: 1 vs. 50 cents) × 2 (reward presentation: brief vs. long) within-subjects design, with EEG measurements to test for between-subjects effects. After 20 practice trials, participants completed 160 trials in total, 40 repetitions per condition. Fifty-cent coins were used instead of 10 cent, in an attempt to maximize reward effects on effort as well as on ERN (see Bijleveld et al. 2010, for use of the same coins and masks).

#### Procedure

Upon entering the lab participants signed informed consent and received verbal instructions, after which the facial and EEG electrodes were applied. Participants were seated in front of a 60 Hz CRT monitor with a resolution of 1,024 by 768 at a viewing distance of 60 cm, which was measured at the beginning of each study. Participants were told that on each trial in the study they would see a coin (1 or 50 cents), which they could earn by correctly solving the subsequent task.

#### Experimental task

The task was analogous to the one used in the first study. Again, each trial started with a fixation cross visible for 1,000 ms. Then, participants saw a coin, presented between masks for 17 (suboptimal) or 300 ms (optimal), where the combined presentation time of masks and stimuli was kept constant. After the presentation of the coin, another fixation cross appeared for a random duration between 1 and 3 s. The subsequent task consisted of a field of squares and triangles displayed in a 4 by 4 grid, and participants were to assess whether there was an even or odd number of triangles (actual numbers ranging from 3 to 8). After an answer was provided using either of two keys on the keyboard, a feedback screen appeared indicating whether their answer was right or wrong. The amount of money they received for each correct trial was contingent on their response latency: the faster they were, the more money they earned. The amount was computed as *M* = *V* − (*V* × (*T*/*A*)), where *M* is the amount of money earned, *V* is the value of the coin that was presented (in cents), *T* is the response latency, and *A* is a participant-specific ability parameter that was computed based on participants’ performance during the practice trials. This parameter was computed so that it would roughly force an equal number of errors per participants. It generated a response window within which the reward declined to zero that was so brief that participants had to make an educated guess, rather than engage in actual counting to still be able to earn a percentage of the reward. Under this time pressure, we assumed to find reward effects mainly on accuracy. With reaction times >0, the earned amount was always less than the coins presented at the beginning of the trial. When the response was incorrect, the participant received no money. The specific amount earned per trial with their overall cumulative earnings was shown at the final screen of each trial. Then, a new trial started.

#### Coin visibility

In order to verify whether participants could accurately discriminate between high- and low-value coins when presented for 17 ms, a signal detection task was run after the experimental task on the same participants. They were exposed to the same coin stimuli as in the task (i.e. 1 vs. 50 cents, presented for 17 vs. 300 ms), and they were subsequently asked to indicate the identity of the stimulus using two keys representing the high and low value coin. The task consisted of 120 trials, with 30 trials per condition.

#### EEG recording

EEG was recorded from 32 scalp locations using to the International 10–20 EEG System with Ag–AgCl-tipped electrodes. Electro-oculogram (EOG) was recorded from bipolar montages above and below the right eye and the outer canthi of the eyes. Raw EEG recordings were made with the ActiveTwo system (BioSemi, Amsterdam, The Netherlands) relative to the common mode sense (CMS). All data were recorded with a sampling rate of 2,048 Hz, and data were stored for offline analysis.

#### ERPs

EEG data recorded during the task were re-referenced offline to the averaged signal of all scalp channels, and subsequently filtered with a 1 Hz high-pass filter and a slope of 24 dB/oct, and a 10 Hz low-pass filter with a slope of 24 dB/oct. Data were segmented into 2,500 ms windows with a 100 ms baseline correction with respect to the feedback stimulus onset. Ocular artifacts were corrected using the Gratton and Coles algorithm (Gratton et al. 1983) in addition to a visual inspection and segments containing artifacts were removed (difference criterion between two subsequent data points of 50 μV; differences criterion within segment of 100 μV; absolute amplitude criterion of 50 μV). For each participant the segments containing trials with an erroneous response were combined for the calculation of an average feedback-locked ERN. The average was determined on the FZ electrode within a window of 0–300 ms after the feedback, where the lowest and subsequent highest amplitudes were averaged over miss-trials per participant. Taking the difference between these two peaks acted as an additional baseline correction. These amplitudes were later on subtracted, highest minus lowest, to end up with an overall measurement of individual ERN.

### Results and discussion

#### Coin visibility

To assess whether participants could consciously discriminate between high and low rewards for suboptimal cue trials, forced-choice signal detection test data were analyzed using a binomial test. This revealed an accuracy of 54.41 % (*SD* = 10.49), slightly but significantly above chance with *z* = 2.60, *p* < .01. Accuracy was significantly lower, though, than for optimally presented cues (*M*_optimal_ = 96.07 %, *SD* = 18.52 %), *t*(28) = −10.87, *p* < .001.

#### Counting task

The mean accuracy scores were subjected to an analysis of variance, with reward value (low vs. high) and cue presentation (suboptimal vs. optimal) as within-participants variables. This analysis revealed a significant main effect of reward value, *F*(1, 27) = 13.89, *p* < .001, η_*p*_^2^ = .34, with no effect for cue presentation, nor an interaction effect (both *F*’s < 1). The effect of reward value was significant for suboptimal cues (*M*_high_ = 85.71 %, *SD* = 9.47 %; *M*_low_ = 81.43 %, *SD* = 7.31 %), *t*(28) = 2.52, *p* = .01 (one-tailed), but not for optimal cues (*M*_high_ = 85.89 %, *SD* = 7.79 %; *M*_low_ = 82.68 %, *SD* = 10.43 %), *t*(28) = 1.67, *p* = .06 (one-tailed). In addition, the mean response times were also subjected to a similar analysis of variance. This analysis revealed no significant main effect of reward value, *F* < 1; but did show an effect for Cue presentation, *F*(1, 27) = 5.10, *p* = .03, η_*p*_^2^ = .16. Although the interaction was not significant, *F*(1, 27) = 3.85, *p* = .06, η_*p*_^2^ = .13, reward effects were explored for suboptimal and optimal reward cues separately. Participants did not respond faster on high reward compared to low reward trials (*M*_high_ = 2,060 ms, *SD* = 385; *M*_low_ = 2,044 ms, *SD* = 361) for suboptimal cues, *t* < 1, but did so for optimal cue trials (*M*_high_ = 2,002 ms, *SD* = 339; *M*_low_ = 2,049 ms, *SD* = 346), *t*(28) = 1.97, *p* = .03 (one-tailed).

With our signal detection scores slightly above chance, we investigated the relation between coin visibility and the reward effect on accuracy for brief presentations using the method recommended by Greenwald et al. (1995). The reward effect was regressed on the coin visibility variable, which was recoded so zero represented chance level detection. It was found that the slope of the regression line was not significant, *b* = .15, *t*(26) = 0.81, *p* = .43, indicating no relation between conscious detection of the reward cue and the reward effect. Then we tested whether the intercept was significantly above zero, *b* = .04, *t*(26) = 2.02, *p* = .03 (one-tailed), which it was. Hence, the regression model suggests a reward effect even when reward detection is at chance level. Together, this suggests that increases in the reward effect for suboptimal reward cues are not dependent on conscious awareness.

#### ERN

**Fig. 2:**
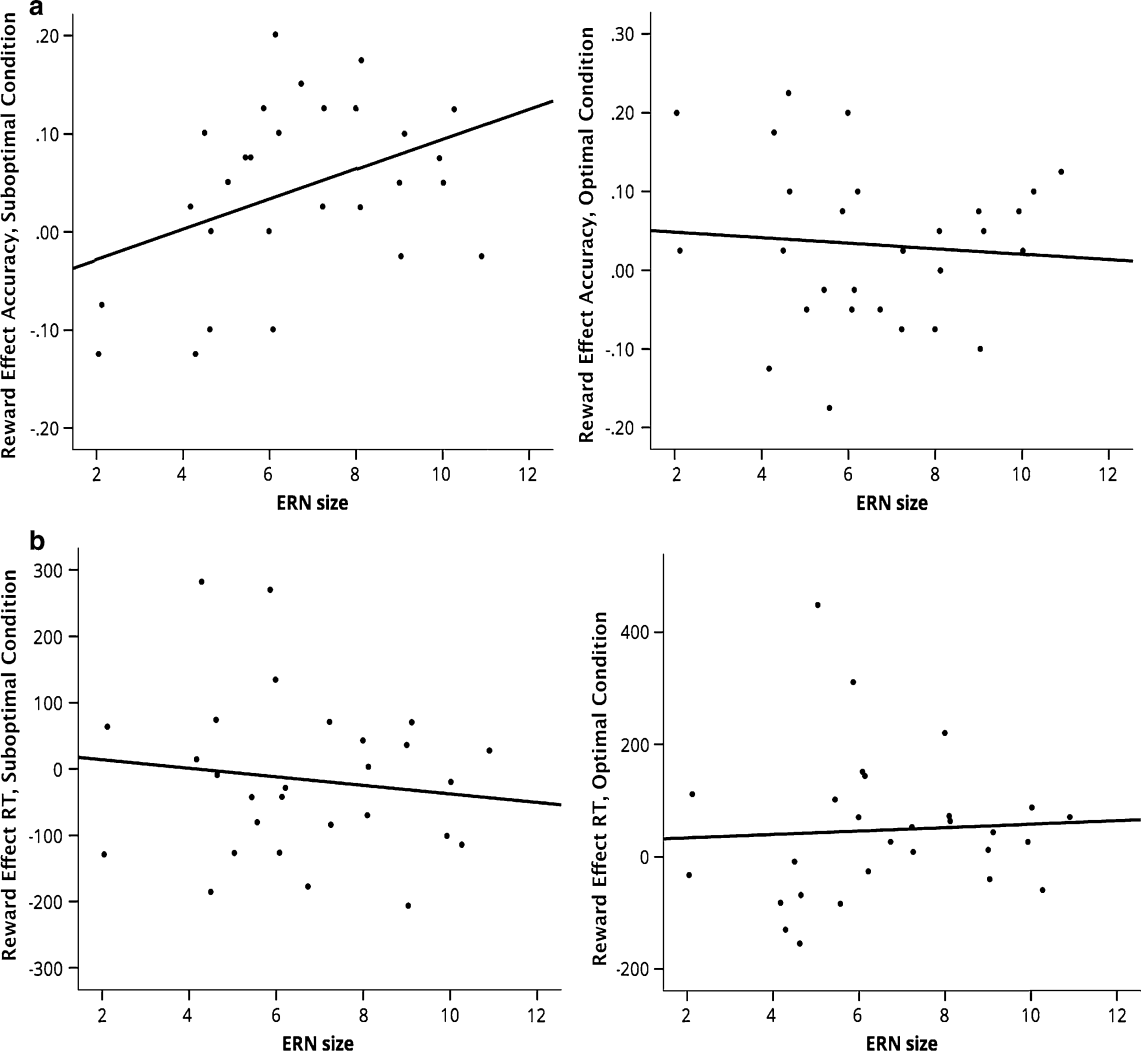
Study 2: Scatterplots of the reward effect against ERN size in both the suboptimal condition (*left*) and the optimal condition (*right*), for accuracy and response times (performance on low reward trials subtracted from high reward trials). **a** Accuracy, **b** response times

To test the degree to which the effects of optimal and suboptimal reward cues were modulated by dopaminergic functioning, we computed individually calculated ERN size, by averaging ERN size over all incorrect trials. First a Pearson correlation was computed between averaged ERN and the reward effect on accuracy (accuracy for low reward cues subtracted from that for high reward cues). As expected, ERN size correlated positively with the reward effect for suboptimal reward cues, *r*(28) = .40, *p* = .02 (one-tailed), but not with that for optimal reward cues, *r*(28) = −.08, *p* = .68 (see Fig. 2). These two correlations are significantly different from each other, with a Steiger’s Z score of 1.80, *p* = .02 (one-tailed; Steiger 1980). ERN size did not significantly correlate with the reward effect on response times for suboptimal, neither for optimal reward cues, *p*’s > .05. To control for potential tradeoffs between speed and accuracy, we partialed out the effects of reaction times on accuracy (see Custers and Aarts 2003 for a similar analysis). ERN size remained significantly correlated with the reward effect for brief reward cues, *r*(25) = .39, *p* = .02 (one-tailed).

#### Discussion

Looking at cognitive effort responses, we replicated our earlier findings and demonstrated that participants performed better when presented with a reward cue of higher value. Although there was a main effect of reward value on accuracy, there was no significant difference in this effect for the different cue presentations. The analyses of the reaction times suggest that this may be due to a shift in emphasis on reaction times on trials with optimal reward cues. Similar strategic shifts were observed by Bijleveld et al. (2010), although in that particular paradigm participants slowed down their responses in order to increase accuracy. The fact that we observe a speedup here with no significant effect on accuracy for optimal trials is probably due to the short time window in this task, which forces participants to make an educated guess. Under these circumstances, slowing down would probably not lead to a reliable increase in accuracy.

Overall, higher accuracy was linked to increased striatal dopaminergic functioning, as indicated by averaged ERN amplitude, but only for suboptimal reward cue trials. In addition, this effect was still present when controlling for speed, which shows that the accuracy effect is a genuine effect on performance and not caused by a speed accuracy trade-off.

According to the signal detection task participants were able to discriminate the briefly presented cues slightly above chance level. However, there are several reasons to assume that effort responses on the brief reward cue trials do not reflect responses that are the result of deliberate reward processing. First of all, there seemed to be a difference in the effects on reaction times for suboptimal and optimal reward cues. Although the interaction was not significant, participants were faster for high than low optimal reward cues, which may reflect strategic behavior as faster reaction times were rewarded. No such effect was observed for brief reward cues. Second, in line with our predictions, ERN only correlated with the reward effect for brief reward cues, which suggest that for these cues, the effect was not overruled or overshadowed by strategic reactions or reflections on reward value. As such, we strongly suspect that despite reward detection being slightly above chance for brief reward cues, responses to these cues could still be regarded as reflecting initial reward processing.

One could argue that at first sight, measuring of ERN on the same trials that serve as performance measure presents a confound. That is, an increase in accuracy (less errors) would cause ERNs to be calculated over fewer trials, possibly increasing the resulting mean ERN as errors would be rarer. However, if this would have caused the effect, one would expect performance to be correlated to ERN on trials with optimal reward cues as well, as they would contribute equally to overall accuracy as suboptimal reward cue trial would. Although investigating the relation between ERN and accuracy in each cell of the design would be informative in many respects, the low number of error trials contributing to the ERN average prevented us from running such analyses.

## General discussion

The present research aimed to examine the role of the mesolimbic dopamine system in producing rudimentary effort responses. First of all, our studies replicate earlier findings by demonstrating that effort responses can result from reward cues that are presented suboptimally, which makes the contribution of conscious, deliberative processes unlikely. Second, reward responses were found to be correlated with both resting-state EBR and averaged ERN amplitude—both markers of striatal dopaminergic functioning, but only for suboptimal reward cues. As such, the current findings provide support for the involvement of the mesolimbic dopamine system in effort responses to suboptimal reward cues.

The results presented here support the model of reward pursuit put forward by Bijleveld et al. (2012b), in which effort responses to optimal and suboptimal reward cues are assumed to rely, at least partly, on different anatomical structures in the brain. Whereas processing of suboptimal reward cues is thought to require no awareness and to rely on subcortical brain structures—most notably the striatum—optimal reward cues enjoy full reward processing associated with conscious awareness, which allows for a host of strategic and reflective processes that are supported by higher-level cortical areas. The observation that a correlation between effort responses and striatal dopaminergic functioning was obtained only for suboptimal reward cues fits the notion that for optimal reward cues, initial effort responses produced by rudimentary reward processing can be overruled or overshadowed by these strategic processes.

The finding that individual differences in striatal dopaminergic activity were linked to effort responses solely for suboptimal cues suggests that effects of optimal reward cues rely more on the interaction between these sub-cortical areas and higher cortical processes (Cools 2011). In situations where the task constrains strategic responding, or performance does not benefit from it, initial and full reward processing may produce the same outcomes (see e.g., Bijleveld et al. 2009; cf., Pessiglione et al. 2007). As noted earlier, in previous work we found that people deliberately sacrifice speed for accuracy for when a valuable reward was at stake, causing full reward processing to diverge from the initial course (Bijleveld et al. 2010). Such responses to fully processed reward cues may in this case be very well correlated with individual differences that affect strategic responding independently of striatal dopamine functioning.

This is not to say that differences in performance between suboptimal and optimal reward cues necessarily reflect a difference in effort expenditure. As previous research has pointed out (Gendolla et al. 2012), the relation between effort and performance is not always a direct one. Strategic processes on trials with optimal reward cues could either change the relation between effort and performance, or affect the process of effort expenditure by themselves. In any case, because of the observed correlation between striatal dopamine functioning and performance for suboptimal reward cues, we assume that performance measures are a better indicator of effort expenditure in the case of suboptimal than optimal reward cues.

Although Pessiglione et al. (2007) ruled out strategic responding as suboptimal reward cues could not be consciously detected above chance level, the effect on behavior under these conditions was found to be unrelated to striatal activity. A possible reason for this may be that the activity occurs in a quicker and more transient way compared to optimal reward cues. Such an explanation is in line with the idea that conscious awareness of a stimulus keeps information carried by the stimulus active over a sustained period of time (Dehaene and Naccache 2001). If true, this accounts for why a direct link between suboptimal reward cues and striatal activation was not established, as it may be the case that ventral striatum activation occurred too quickly and too transiently to be detected with fMRI. The current research is not hindered by these methodological constraints as it relied on individual differences in striatal dopaminergic functioning in relation to overall task performance.

Although our results are consistent with our theoretical predictions, the correlational nature of our findings may allow for alternative accounts. Most notably, previous studies have demonstrated relationships between activation in the ventral striatum and individual differences in learning performance (Santesso et al. 2008; Schonberg et al. 2007; Vink et al. 2013), and learning effects might explain performance in our task without having to assume direct involvement of the dopamine system in producing the effort response. That is, experimental tasks designed to study the effects of optimal versus suboptimal reward cues involve feedback about the obtained reward. This feedback, in turn, provides a clear opportunity for basic stimulus–response learning. So, it could be argued that learning takes place only on trials with optimal, consciously processed reward cues. Effort responses to suboptimal reward cues, then, could just be learned, a-motivational responses. The present pattern of findings, however, seems incompatible with this account, as the correlations between markers of dopaminergic functioning and performance proved only to exist for suboptimal rewards. So, if anything, it seems that the dopaminergic system is involved in shaping performance especially on these trials. Nevertheless, direct evidence for this involvement is rather scarce (but see Pessiglione et al. 2008, 2011). More research is thus needed to delineate the role of the subcortical reward center in the processing of suboptimal reward cues.

The present research is in line with previous research (Bijleveld et al. 2012b), suggesting that people are able to pursue rewards that are perceived without awareness, and that reward pursuit in this case is guided by a subcortical reward system that is different from the one that dominates conscious reward pursuit. The fact that such a difference was observed in our second study even though brief reward cues were not fully shielded from conscious detection suggest that differences in conscious awareness may by associated with the operation of these different types of reward processing, but that conscious awareness itself is perhaps not the crucial factor that distinguishes them.

The general observation that different mechanisms may underlie the processing of optimal and suboptimal reward cues has larger implications for many aspects of cognition and behavior. The rudimentary reward system has been shown to play a role in processes ranging from goal pursuit (Custers and Aarts 2010), the experience of agency (Aarts et al. 2009), and financial decision-making (Knutson and Greer 2008), to the experience of intrinsic enjoyment during task performance (Murayama et al. 2010). In addition, variations in dopaminergic activity have been linked to various neurological and psychiatric disorders, including Parkinson’s disease, schizophrenia, depression, and drug addiction (Salamone et al. 2005). Exploring the functional processes affected by these conditions can help in understanding the scope of the dysfunction, and how far its symptoms reach in day-to-day functioning. Taken together, by distinguishing between two different mechanisms for reward processing, the present approach to studying motivated action may in the future prove fruitful for enhancing our understanding of human behavior across a wide variety of domains.
